# Small extracellular vesicles derived from four dimensional-culture of mesenchymal stem cells induce alternatively activated macrophages by upregulating IGFBP2/EGFR to attenuate inflammation in the spinal cord injury of rats

**DOI:** 10.3389/fbioe.2023.1146981

**Published:** 2023-04-28

**Authors:** Junhua Wang, Qingshuai Wei, Yue Yang, Mingtian Che, Yuanhuan Ma, Lizhi Peng, Haiyang Yu, Huijuan Shi, Guanheng He, Rongjie Wu, Ting Zeng, Xiang Zeng, Wenbin Ma

**Affiliations:** ^1^ MOE Key Laboratory of Gene Function and Regulation, State Key Laboratory of Biocontrol, School of Life Sciences, Sun Yat-sen University, Guangzhou, China; ^2^ Key Laboratory for Stem Cells and Tissue Engineering, Ministry of Education, Sun Yat-sen University, Guangzhou, China; ^3^ Biobank and Pathology Shared Resources, Cedars-Sinai Medical Center, Los Angeles, CA, United States; ^4^ Guangzhou Institute of Clinical Medicine, Guangzhou First People’s Hospital, South China University of Technology, Guangzhou, China; ^5^ Department of Orthopedics, Guangdong Provincial People’s Hospital, Guangdong Academy of Medical Sciences, Guangzhou, China; ^6^ Department of Acupuncture, The First Affiliated Hospital, Sun Yat-sen University, Guangzhou, China; ^7^ Sun Yat-sen Memorial Hospital, Sun Yat-sen University, Guangzhou, China; ^8^ Lab of Stem Cell Biology and Innovative Research of Chinese Medicine, National Institute of Stem Cell Clinical Research, Guangdong Provincial Hospital of Chinese Medicine/Guangdong Academy of Chinese Medicine/The Second Affiliated Hospital of Guangzhou University of Chinese Medicine, Guangzhou, China

**Keywords:** spinal cord injury, four dimensional culture, Small Extracellular Vesicles, macrophage polarization, IGFBP2/EGFR

## Abstract

Effectively reducing the inflammatory response after spinal cord injury (SCI) is a challenging clinical problem and the subject of active investigation. This study employed a porous scaffold-based three dimensional long-term culture technique to obtain human umbilical cord mesenchymal stem cell (hUC-MSC)-derived Small Extracellular Vesicles (sEVs) (three dimensional culture over time, the “4D-sEVs”). Moreover, the vesicle size, number, and inner protein concentrations of the MSC 4D-sEVs contained altered protein profiles compared with those derived from 2D culture conditions. A proteomics analysis suggested broad changes, especially significant upregulation of Epidermal Growth Factors Receptor (EGFR) and Insulin-like Growth Factor Binding Protein 2 (IGFBP2) in 4D-sEVs compared with 2D-sEVs. The endocytosis of 4D-sEVs allowed for the binding of EGFR and IGFBP2, leading to downstream STAT3 phosphorylation and IL-10 secretion and effective induction of macrophages/microglia polarization from the pro-inflammatory M1 to anti-inflammatory M2 phenotype, both *in vitro* and in the injured areas of rats with compressive/contusive SCI. The reduction in neuroinflammation after 4D-sEVs delivery to the injury site epicenter led to significant neuroprotection, as evidenced by the number of surviving spinal neurons. Therefore, applying this novel 4D culture-derived Small Extracellular Vesicles could effectively curb the inflammatory response and increase tissue repair after SCI.

## 1 Introduction

Spinal cord injury (SCI) is detrimental to the central nervous system and often results in lifelong sensory and motor impairments (Rahimi-Movaghar et al., 2013; Silva et al., 2014). The hostile microenvironment caused by secondary injury is dominated by chronic inflammation and imposes great challenges to the structural and functional reconstruction of the injured spinal cord (Ronaghi et al., 2010; Zhang et al., 2012). Therefore, effectively curbing the post-injury inflammatory response is significant to reconstructing a pro-regenerative niche for spinal cord tissue repair. Studies have shown that transplantation of MSCs or their secreted sEVs in the injured area could directly affect immune regulation (Liu et al., 2014; Sun et al., 2018; Liu et al., 2019; Hade et al., 2021) and reduce neuroinflammation associated with SCI (Spaggiari et al., 2009; Glenn and Whartenby, 2014; Urdzikova et al., 2014). Specifically, sEVs/exosome transplantation surpasses cell transplantation in host acceptance, donor fate determination, and batch-to-batch consistency (He et al., 2018; Kalluri and LeBleu, 2020), making it a promising material for tissue repair. Indeed, there are growing evidences suggesting the therapeutic effects of sEVs on various disorders with the potentials for clinical application (Bahmani and Ullah, 2022). As natural nano-scale vesicles, sEVs have many advantages compared with other engineered nanomaterials. However, whether transplanted MSC-derived sEVs embrace the critical elements required to tackle the complex post-SCI microenvironments remains to be explored.

Previous studies have developed a three dimensional (3D) culture methodology for constructing neural tissue or spinal cord-like tissue derived from adult stem cells in a trophic factor-defined 3D scaffold. With the dynamic interactions between stem cells and the ambient 3D microenvironment over time, stem cells can recapitulate developmental scenarios and develop into homeostatic entities resembling critical tissue features such as functional cells and their extracellular matrix (ECM). For example, seeding neural stem cells (NSCs) in a sustained neural trophic factor (NT)-3 releasing bioscaffold resulted in the formation of a self-organized neural network tissue with mature neuronal function (Li et al., 2021). This proof-of-concept study suggests that by allowing sufficient interactions between the stem cells and the microenvironment, a single neural trophic factor (NT-3) may be enough to support neurogenesis with mature neuronal function and efficacy in treating SCI. Additionally, following the dynamic interactions between the constructed stem cell-derived white matter-like module and the grey matter-like module over some time (∼14 days), the modular assembled spinal cord-like tissue resembled a more mature neuronal and glial structure and function (Lai et al., 2018). Taken together, this evidence suggests that the self-organization of stem cells during 3D culture confers a homeostatic niche favorable for cell survival and function, a principle in “4D” biology highlighting the “time” factor during 3D culture (Tibbitt and Anseth, 2012). Given their significant role in intercellular communication, it was hypothesized that the sEVs derived from the 3D culture of MSCs over time (i.e., the 4D culture) might recapitulate critical elements essential for a homeostatic microenvironment. However, the exact contents of the sEVs derived from 4D culture of MSCs and their function in tissue repair have never been studied before. Whether sEVs derived from 4D culture of MSCs may exhibit stronger pro-homeostasis/tissue repair efficacy for SCI treatment and the underlying mechanism is to be determined.

In this study, for the first time we characterized the main features of sEVs derived from the 4D-culture of MSCs (four dimensional culture-derived sEVs, 4D-sEVs) and investigated their effect on regulating the inflammatory response in the injured area of the spinal cord. The results suggested a potent anti-inflammatory effect of the 4D-sEVs. This feature may enable 4D-sEVs use in future clinical applications for SCI patients.

## 2 Materials and methods

### 2.1 Acquisition and purification of four dimensional-derived sEVs

As previously described (Zeng et al., 2015; Yang et al., 2021), a three-dimensional (3D) gelatin sponge scaffold with a diameter and length of 4 mm was prepared and fully hydrated overnight in a D-hanks solution. The scaffolds were seeded with 1 × 10^5^ hUC-MSCs, and ten scaffolds/well were cultured in a six-well plate (3D-scaffold group). Ten cell-loading scaffolds were cultured in 10 mL culture medium in each well of a six-well plate. Equal numbers of hUC-MSCs were plated in cell culture flasks (this is the 2D group). The two groups were incubated at 37°C for 14 days, and the culture medium was changed every 2 days. After 14 days, the growth medium was removed, cells were washed 3 times with D-hank’s, and then serum-free hUC-MSC sEVs secretion-promoting medium Ultra CULTURETM (BP12-725F, Lonza, Basel, Switzerland) was added to the cells, and the supernatant was collected after 48 h.

The collected sEVs were purified using centrifugation, adapted from a previous protocol (Thery et al., 2006). The supernatant (150 mL) was centrifuged at 300 *g* for 10 min, and then the supernatant was collected and filtered using a 0.22 μm filter. Centrifugation was performed at 2000×g for 10 min and 10,000×g for 30 min to remove cell debris. Next, We used overspeed centrifuge (Optima XE 100, Beckman, United States) with the rotor of type 70 Ti and the k-factor of 44 to extract extracellular vesicles. Centrifugation was performed at 100,000×g (37000 RPM) for 90 min, the supernatant was removed, and the pellet was resuspended in PBS. Finally, centrifugation was performed again at 100,000×g for 90 min, and the pellet was resuspended in 200ul PBS and then stored at −80°C.

The yield of sEVs/per cell was calculate by dividing the number of sEVs by the total cell number of MSCs within the scaffolds following 4D culturing. The cell number in each scaffold was numerated after detaching them with enzymes (0.25% trypsin and 2 mg/mL type I collagenase, 1:1).

### 2.2 Characterization of sEVs

The morphology of the sEVs was determined by Transmission Electron Microscopy (TEM). Fifty (50) μg of sEVs were diluted (1:40) with PBS, then diluted sEVs suspension (20 μL) was dropped on a 300-mesh cell strainer for 10 min. Excess liquid was absorbed with filter paper, counterstained with 1% phosphotungstic acid for 30 s, and observed under a transmission electron microscope.

We used Nanoparticle Tracking Analysis (NanoSight NS300, Malvern,United Kingdom) with the particle concentration of 10^7^–10^9^ and the maximum diameter of 2 um to detect the particle number and size distribution of sEVs. Samples were diluted (1:1,500) with PBS, injected into the device manually, we set the threshold at 5 and videos were acquired at ambient temperature (camera level 9) for 1 min per sample.

sEVs were diluted with PBS at 1:10, 1:50, 1:100, and 1:200, respectively. The protein standards and sEVs samples were added to a 96-well plate according to the BCA Protein Concentration Assay Kit instructions (P10010S, Beyotime, Shanghai, China), and the absorbance was measured at a wavelength of 562 nm to detect protein concentration of sEVs. Therefore, the protein concentration for each particle could be calculated by dividing the total protein concentration by the particle number of each sample.

### 2.3 sEVs labeling

Under sterile conditions, the sEVs (100 μg) were resuspended in PBS (1 mL), and 10 µL Dil Cell-Labeling Solution (V22885, ThermoFisher, Waltham, MA, United States) was added to the sEVs suspension. After gently blending, the suspension was incubated at 37°C for 10 min. Then, the dye-labeled sEVs were ultracentrifuged at 100,000×g for 60 min. Lastly, the supernatant was discarded, and PBS (200 µL) was added to resuspend the pellet.

### 2.4 Transfection of siRNA

The hUC-MSCs (1 × 10^6^) were seeded in 6-well plates. After 24 h, siRNA (Ge-nomeditech, Shanghai, China) and Lipofectamine RNAiMAXTM (2276055, ThermoFisher, Waltham, MA, United States) diluted with OPti-MEM (2323655, ThermoFisher, Waltham, MA, United States) were mixed in a 1:1 ratio. After 20 min at room temperature, siRNA and Lipofectamine RNAiMAXTM were added to the cell culture medium and incubated for 6 h. After 6 h, a culture medium without siRNA was added, and cell RNA and protein were extracted after 24 h. In addition, hUC-MSCs (1 × 10^5^) were seeded on three-dimensional gelatin sponge scaffolds, and 10 scaffolds were co-cultured in 6 well plates. The siRNA transfection was performered according to the steps listed above, and the transfection was repeated every 3 days. After 14 days, the RNA, proteins, and sEVs were extracted.

### 2.5 Preparation of the RAW264.7 cells *in vitro*

Cryopreserved RAW264.7 macrophages purchased from ATCC (Manassas, VA) were cultured in a high-glucose DMEM medium containing 10% fetal bovine serum (FBS). The cells were cultured at 37°C, and the medium was changed every 3 days when the cell confluence was approximately 80%.

The RAW264.7 cells were seeded into 24-well plates at 1 × 10^5^ cells/well, and divided into 6 groups: Control group, LPS group, 2D-sEVs group, 4D-sEVs group, IGFBP2-siRNA-#2 and NSC74859 group. First, the LPS group, 2D-sEVs group, 4D-sEVs group, IGFBP2-siRNA-#2 and NSC74859 group were stimulated with 100 ng/mL lipo-polysaccharide (LPS, Sigma-Aldrich, Waltham, MA, United States) for 12 h, and the Control group contained an equal volume of medium without LPS. Next, the supernatant was removed and washed 3 times with PBS. STAT3 inhibitor, S3I-201(NSC74859, S1155; Shanghai, China) is used as described in the article (Zheng et al., 2019). NSC74859 group was pre-treated with S3I-201 (10 µM) for 1 h. Dil dye-labeled 2D sEVs (40 μg/well) were added to the 2D-sEVs group, and an equal amount of Dil-labeled 4D sEVs/IGFBP2-siRNA-#2-4D sEVs were added to the 4D-sEVs/NSC74859/IGFBP2-siRNA-#2 group for 24 h. Dil dye was added to the Control and LPS group for 24 h. The superna-tant was collected, and the cells were fixed for immunohistochemical staining.

### 2.6 Establishment of the rat spinal cord injury model and sEVs injection

All animal protocols were approved by the Animal Care and Use Committee of Sun Yat-sen University. Adult female SD rats (weighted 220–250 g) were provided by the Experimental Animal Center of Sun Yat-sen University. All animals received an intraperitoneal injection of 1% sodium pentobarbital (40 mg/kg) for anesthesia. After skin preparation, disinfection and fixation, a laminectomy was performed to expose the T9–T10 segments. A pressure device with a mass of 50 g was placed at the T10 segment for 10 min with a fixed spinous process. Then, we staunched the bleeding and sutured it layer-by-layer. 48 h later, a second operation was performed. After anesthesia, the pia mate was cut open, and the injured site of the spinal cord was gently flushed with saline to remove necrotic tissue. GelMA (20%) hydrogel (SE-3DP-0205, StemEasy, Jiangyin, China) and 2.5% LAP (SE-3DP-0105, StemEasy, Jiangyin, China) were mixed in a 1:1 ratio, and Dil dye-labeled 2D/4D sEVs (500 μg) were resuspended in the mixture. Injected with number of EVs corresponding to 500ug protein for each rat was (13.33 ± 3.50) x 10^11^ (2D-sEVs) and (6.34 ± 0.63) x 10^11^ (4D-sEVs) (Supplementary Figure S2L). Next, the mixture was injected into the syringomyelia with a microsyringe and irradiated with a 405 nm UV lamp for 10 s. At the same time, the SCI group only injected pure Dil dye and hydrogel, and the sham group only opened the lamina without crushing the spinal cord. When the GelMA hydrogel containing sEVs solidified, an incision was sutured layer-by-layer. All rats received post-operative care, including intramuscular injection of penicillin (50,000 U/kg per day) for 3 days and manual emiction two times daily until their automatic micturition function was reestablished.

### 2.7 Tissue preparation, immunohistochemical/neutral red staining and neuron counting

Ten (10) days after the surgery (i.e., 7 days after sEVs delivery), the animals were anesthetized and transcardially perfused with 4% paraformaldehyde in 0.1 M phosphate buffer. Then, spinal cords were removed, post-fixed, and dehydrated in sucrose/PB (30%). Longitudinal cryosections of the spinal cord, including the injury site, were cut at 20 μm and mounted on slides. *In vitro*, after removing the medium from the well plate, it was fixed with 4% PFA overnight and then washed three times with PBS. Immunohistochemistry was performed as described below: Briefly, the sections were rinsed with PBS, blocked with 10% normal goat serum, and 0.3% Triton X-100 in 0.01 M phosphate-buffered saline for 30 min at 37°C, and incubated with primary antibodies overnight at 4°C. Subsequently, the sections were rinsed with PBS and incubated with a secondary antibody for 1 h at 37°C. Finally, the sections were rinsed with PBS, coverslipped, and examined under a confocal microscope.

The slides were dried and then soaked in Neutral Red staining solution (C0123, Beyotime, Shanghai, China) for 20 min, distilled water for 10 s, alcohol gradient decolorization for 5 s each 70%, 80%, 95%, and 100%, xylene transparent twice (each 4 min), and neutral resin sealing.

For each group, the five transverse sections (5 rats/group) in the L1 segment of the spinal cord were selected by the method of one of ten. After neutral red staining, images were taken under light microscope (100x) and neurons of Clarke’s nuclei (CN) on 5 serial spinal cord sections of each rat were also counted (only neurons with nuclei were counted). There were five rats in each group.

### 2.8 Morphological quantification

The quantitative analysis of CCR7, CD206, p-EGFR and p-STAT3-positive cell *in vivo* was performed by Laser Scanning Confocal Microscope (LSM800, Carl Zeiss AG) and ZEN 2.3 software (Carl Zeiss AG). One in every 10 longitudinal sections from each rat was processed (5 rats/group). After immunostaining with the respective markers, four randomized fields (200×) within the areas of the injury/graft site of the spinal cord were chosen for imaging and analysis. Immunofluorescence probing cells with identifiable nuclei (counterstained with nuclear dye Hoechst33342) were deemed immunopositive-stained cells. The percentage of CCR7, CD206, p-EGFR and p-STAT3-positive cells was calculated by dividing the number of double labeled positive cells (CCR7^+^/CD68^+^, CD206^+^/CD68^+^, p-EGFR^+^/CD68^+^, p-STAT3^+^/CD68^+^) by the total number of CD68-positive cells. *In vitro*, the round coverslip with cells evenly distributed was divided into four quadrants, and a randomized field (200×) in each quadrant was selected for imaging and analysis. Triplicates were made for each study and the independent experiments were repeated for 4 times, and the positive percentage of cells was calculated by the same method *in vivo*.

### 2.9 Co-immunoprecipitation and Western blot (WB) analysis

RAW264.7 cells (1 mL/10^7^ cells) were lysed with pre-cooled (4°C) RIPA Buffer (P0013, Beyotime, Shanghai, China) supplemented with protease (CW2200S, CWBIO, Beijing, China) and phosphatase inhibitor (CW2383S, CWBIO, Beijing, China), centrifuged at 14000 *g* for 15 min (4°C), and the supernatant was transferred to a new centrifuge tube. The Protein A+ G Agarose (P2012, Beyotime, Shanghai, China) was diluted twice with PBS. Nex, add 100 μL Protein A + G Agarose beads to 1 mL total protein and shake slowly at 4°C for 2 h. After centrifugation at 14000 *g* for 15 min (4°C), the supernatant was transferred to a new centrifuge tube to remove Protein A + G Agarose. The extracted protein was diluted to 5 μg/µL in PBS, and 500 μL of each protein sample (5 μg/μL) was added to IGFBP2 (1:100), EGFR (1:100) and IgG (1:100), respectively. After incubation for 2 h at room temperature, Protein A + G Agarose was added and arose overnight at 4°C. The supernatant was discarded by centrifugation at 14000 *g* for 5 min (4°C), and the remaining precipitate was washed with 800ul PBS for 3 times. Lastly, it was resuspended with 60ul of loading buffer and boiled for WB detection.

After removing the medium from the well plate, an appropriate amount of lysis buffer (P0013, Beyotime, Shanghai, China) was added to lyse the cells. This solution was then boiled and stored in a −80°C refrigerator for later use. Western blot analysis was performed using the tissue samples for 10 days after surgery (4 rats/group). The spinal cords of 15 rats were immediately removed after deep anesthesia. The spinal cord segment (0.5 cm long) containing the injury/graft site was dissected and homogenized in lysis buffer containing 20 mM Tris, pH 7.5, 150 mM NaCl, 0.2% Triton X-100 and the Complete Mini Protease Inhibitor Cocktail. Equal amounts of proteins (20 μg) were separated by 10% SDS-PAGE and then transferred onto a PVDF membrane. After blocking non-specific binding sites with 5% nonfat milk in TBST (0.5% Tween 20 in TBS) for 1 h at room temperature, the membrane was incubated with primary antibodies overnight at 4°C, respectively. The primary antibodies were detected with an HRP-conjugated secondary antibody and then visualized with an enhanced chemiluminescence (ECL) western blot substrate kit (BL523A, biosharp, Beijing, China).

### 2.10 Enzyme-linked immunosorbent assay (ELISA)

After surgery, proteins were extracted from the spinal cord segments (0.5 cm) containing the injury site by ultrasonication (5 rats/group). The protein extract was ob-tained by centrifugation for 20 min at 12,000×g at 4°C. *In vitro*, the supernatant was collected from stimulated RAW264.7 cells. The inflammatory cytokines in the super-natant were measured using ELISA kits (IL-10, TNF-α, Boster Bio, Wuhan, China) according to the manufacturer’s instructions. The values of each protein detected in samples were normalized by the total protein value determined by the bicinchoninic acid method.

### 2.11 Proteomic analysis

The extracted 2D sEVs and 4D sEVs (n = 3 for each group) were placed in a −80°C refrigerator. Proteomics analysis, including trypsin digestion, liquid chromatog-raphy-mass spectrometry (LC-MS), and data analysis, was performed by Jingjie PTM BioLabs.

Protein Extraction: Sample was sonicated three times on ice using a high intensity ultrasonic processor (Scientz) in lysis buffer (8 M urea, 1% Protease Inhibitor Cocktail). The remaining debris was removed by centrifugation at 12,000 g at 4 °C for 10 min. Fi-nally, the supernatant was collected and the protein concentration was determined with BCA kit according to the manufacturer’s instructions.

Trypsin Digestion: For digestion, the protein solution was reduced with 5 mM dithiothreitol for 30 min at 56 °C and alkylated with 11 mM iodoacetamide for 15 min at room temperature in darkness. The protein sample was then diluted by adding 100 mM TEAB to urea concentration less than 2M. Finally, trypsin was added at 1:50 trypsin-to-protein mass ratio for the first digestion overnight and 1:100 trypsin-to-protein mass ratio for a second 4 h-digestion.

To perform detection and analysis, the tryptic peptides were dissolved in 0.1% formic acid (solvent A) and directly loaded onto a homemade reversed-phase analytical column (25 cm length, 75 μm i.d.). The gradient was comprised of an increase from 8% to 22% solvent B (0.1% formic acid in 90% acetonitrile) over 20 min, 22%–35% in 13 min, and climbing to 80% in 4 min, then holding at 80% for the last 3 min, all at a constant flow rate of 600 nL/min on an EASY-nLC 1000 UPLC system. The peptides were subjected to NSI source followed by tandem mass spectrometry (MS/MS) in Q ExactiveTM Plus (Thermo Fisher Scientific) coupled online to the UPLC. The elec-trospray voltage applied was 2.2 kV. The m/z scan range was 400–1,500 for the full scan, and intact peptides were detected in the Orbitrap at a resolution of 70,000. The peptides were then selected for MS/MS using an NCE setting of 30, and the fragments were detected in the Orbitrap at a resolution of 17,500. The data-dependent procedure alternated between one MS scan followed by 20 MS/MS scans with a 30.0 s dynamic exclusion. Automatic gain control (AGC) was set at 5E4. The fixed first mass was set as 100 m/z.

The resulting MS/MS data were processed using the Maxquant search engine (v.1.5.2.8). Tandem mass spectra were searched against the Homo_sapiens_9606_SP_20191115.fasta database concatenated with the reverse decoy and contaminants database. Trypsin/P was specified as a cleavage enzyme allowing up to 2 missing cleavages. The mass tolerance for precursor ions was set to 20 ppm in the First search and 4.5 ppm in the Main search, and the mass tolerance for fragment ions was set to 0.02 Da. Carbamidomethyl on Cys was specified as fixed modification, and oxidation (M), deamidation (N, Q), and acetylation (protein N terminal) were specified as variable modifications. FDR was adjusted to <1%, and the minimum score for peptides was set to >40. Proteins with a fold change >1.5 and *p* < 0.05 are significantly differen-tially expressed.

### 2.12 Statistical analysis

SPSS 13.0 was used for analysis, measurement data were presented as mean ± standard deviation (x ± s), and a one-way analysis of variance (One-Way ANOVA) was performed to compare between means. If the variances are homogeneous, the LSD method is applied for multiple comparisons of the means between groups; if the variances are not homogeneous, the Kruskal–Wallis test was applied to compare means among groups, and the Dunnett’s T3 method was for pairwise comparisons. *p* < 0.05 means the difference is statistically significant.

## 3 Results

### 3.1 Characterization of four dimensional culture-derived sEVs

**FIGURE 1:**
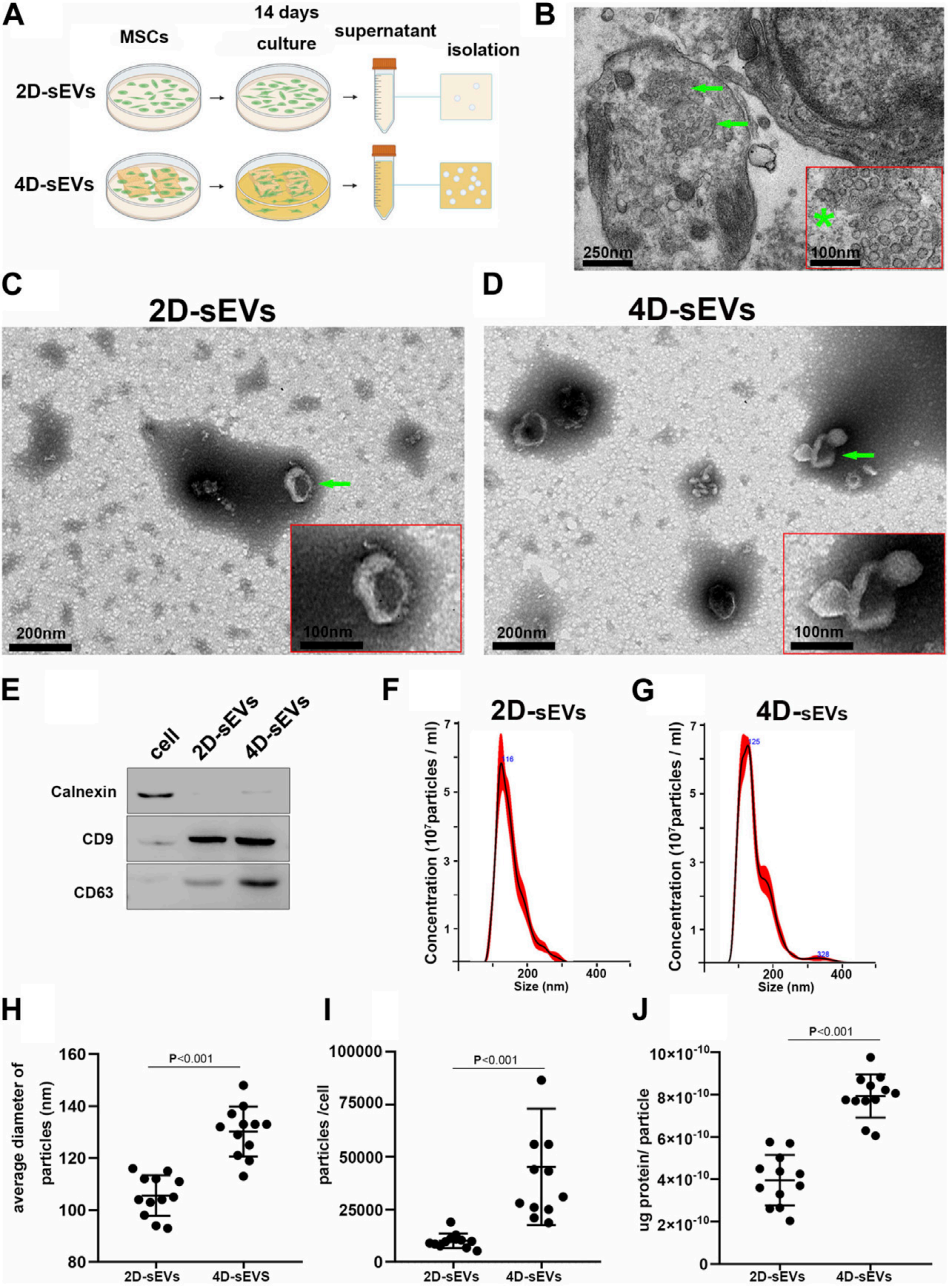
Acquisition and identification of four dimensional culture-derived sEVs (4D-sEVs). **(A)** Flow chart demonstrating the sEVs acquisition process. **(B)** Transmission Electron Microscopy (TEM) images of human umbilical cord mesenchymal stem cells (hUC-MSCs) in tissue-engineered fibroin scaffolds (3D scaffolds). The green arrow indicates exosome-like structures. *: The high magnification of the structure (arrow) of **(B)**. **(C, D)**: TEM micrographs of sEVs. **(E)**: Western blot analysis for sEVs markers (CD9 and CD63). **(F–G)**: Size distribution of sEVs, as evaluated by Nanoparticle Tracking Analysis (NTA). **(H)**: Average sizes of sEVs. **(I)**: Yield of sEVs. **(J)**: Average protein amount of per particle.

The isolation and acquisition process for sEVs is shown in Figure 1A. Using Transmission Electron Microscopy (TEM), exosome-like structures of multiple small vesicles with an average size (∼100 nm) were observed in the cytoplasms of human umbilical cord-derived MSC (hUC-MSC) seeded in three-dimensional (3D) gelatin sponge scaffolds (3D-GS) after 14 days of culture (Figure 1B). After acquisition and purification, the morphology of sEVs secreted by hUC-MSCs in the 2D (2D-sEVs) or 4D (4D-sEVs) cultures had a concave disc-like shape (Figures 1C,D). The biomarkers of sEVs such as CD9 and CD63 in the 2D-sEVs and 4D-sEVs groups were validated by western blot (WB) (Figure 1E). Nanoparticle Tracking Analysis (NTA) showed that the size of the 4D-sEVs (peaked at 125 nm) was larger than that of the 2D-sEVs (peaked at 116 nm) (Figure 1F–H). Additionally, the number of sEVs secreted by a single hUC-MSC following 48 h of serum-free culture in the 4D-sEVs group was approximately 5 times larger compared with the 2D-sEVs group (45280 ± 27725 vs 10100 ± 3,476, *p* < 0.001, Figure 1I). For each sEVs particle, the protein concentration was significantly higher in the 4D-sEVs relative to 2D-sEVs group [(7.94 ± 1.02) x 10^–10^ (ug/particle) vs. (3.96 ± 1.2) x 10^–10^ (ug/particle), *p* < 0.001, Figure 1J]. These results suggest that MSC 4D culture enables a significant increase in sEVs production (including the particle number and protein amount), which may be of great translational value.

### 3.2 Distribution of four dimensional culture-derived sEVs in the injury/injection site of the spinal cord

**FIGURE 2:**
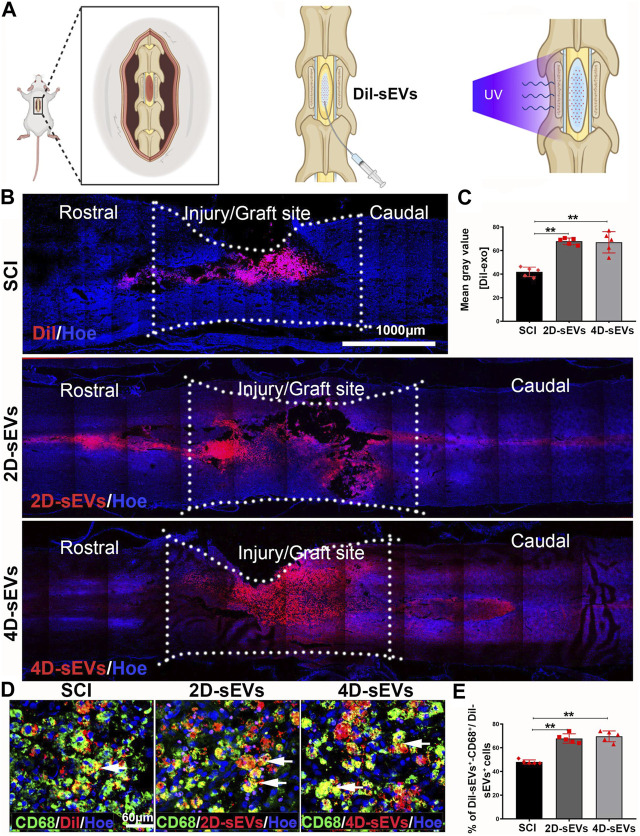
Four dimensional culture-derived sEVs are mainly taken up by macrophages in the in-jury/graft site of the spinal cord. **(A)** Schematic showing how sEVs are injected into the spinal cord. **(B)** Distribution and migration of sEVs in the injury/graft site of the spinal cord visualized using fluorescent images. **(C)** Bar charts showing the mean fluorescence intensity in three groups within the injury/graft site of the spinal cord. **(D)** The fluorescently stained images show that the CD68^+^ cells take up Dil and Dil-sEVs in the injury/graft site of the spinal cord. **(E)** Bar charts showing the ratio of the Dil-CD68^+^ cells to Dil^+^ cells in the injury/graft site of the spinal cord. Statistic difference: **p < 0.01.

A schematic diagram showing the administration of sEVs to the injury site of a spinal cord is shown in Figure 2A. Dil was used to pre-label the sEVs *in vitro* for *in vivo* tracking after injection. After injection into the spinal cord injury site for 7 days, most Dil-labeled sEVs were observed in the injury/injection site, with a small portion in the adjunct area that was rostral/caudal to the injury/injection site (Figure 2B). Quantification of the DiI-labeled area in the injured spinal cord suggested that the SCI with the Dil dye injection group (SCI) had a lower fluorescence intensity compared with the 2D-sEVs injection (2D-sEVs) or 4D-sEVs injection (4D-sEVs) groups. However, there was no significant difference in the fluorescence intensity between the 2D-sEVs and 4D-sEVs groups (Figure 2C). This result suggests that the sEVs could remain in the in-jured site of the spinal cord for at least 7 days.

To further determine the type of cells engulfing sEVs in the injury/injection site of the spinal cord, several markers were used to identify the type of DiI^+^ cells. Immunofluorescence results showed that many DiI^+^ cells were co-labeled with CD68^+^, a marker for activated macrophages/microglia (Figure 2D). Only a tiny portion of Dil^+^cells expressed neural markers such as GFAP (astrocytes), Tuj1 (neurons), or Nestin (neural stem cells) (Supplementary Figure S1A–C). Quantification of the DiI^+^ cells showed that 69.6% ± 4.56% or 67.8% ± 3.96% of Dil^+^ cells were CD68^+^ macrophages/microglia in the 4D-sEVs or 2D-sEVs groups, respectively (*p* > 0.05, Figure 2E). These results show that sEVs were mainly engulfed by CD68^+^-activated macrophages/microglia in the injured spinal cord, and there was no statistical difference in the percentage of DiI^+^ macro-phages/microglia between the 4D-sEVs or 2D-sEVs groups.

### 3.3 Four dimensional culture-derived sEVs polarize the macrophage/microglia phenotype from M1 to M2 to reduce neuroinflammation and protect neurons in the injured spinal cord

**FIGURE 3:**
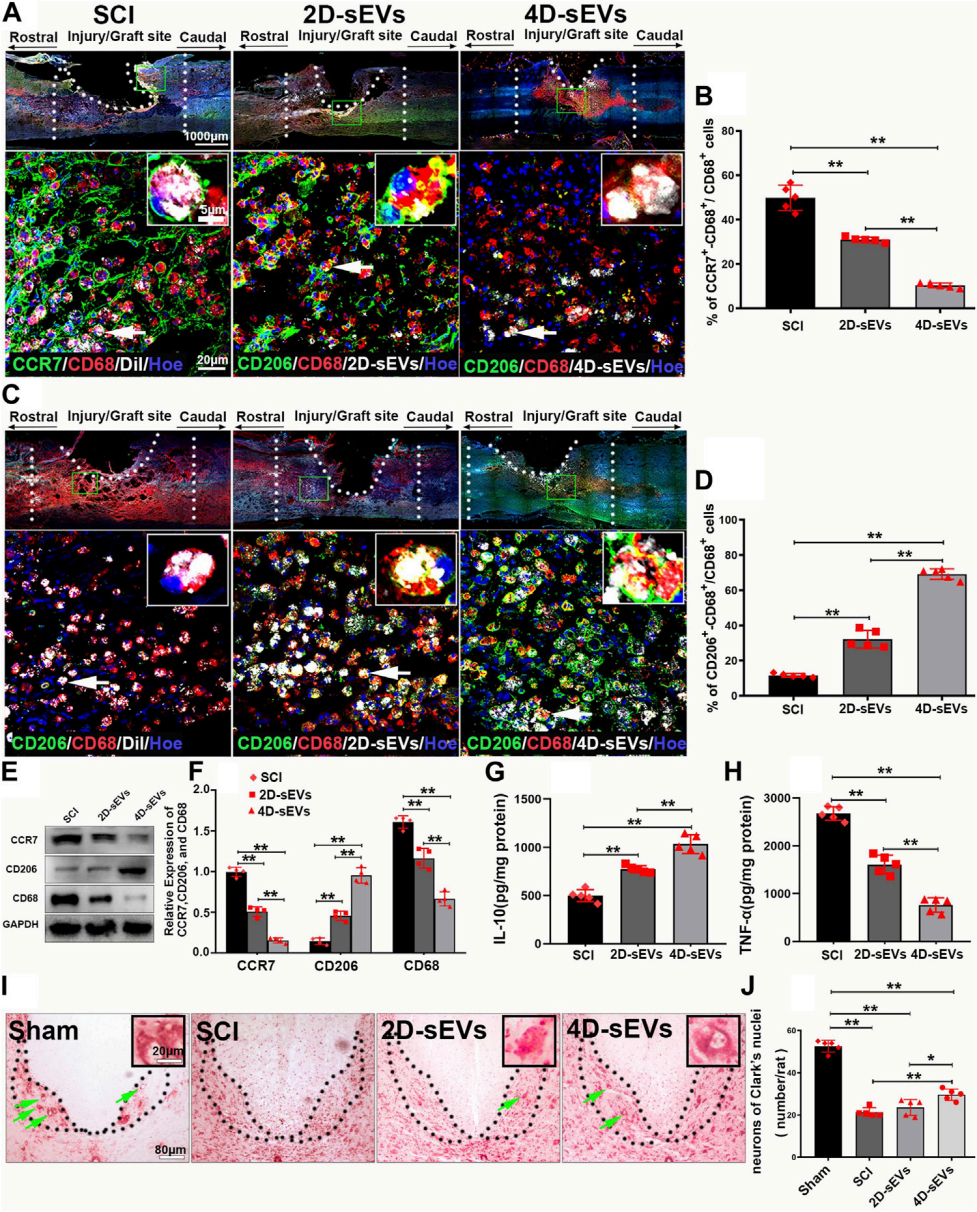
Four dimensional culture-derived sEVs polarized the macrophage/microglia phenotype from M1 to M2 to reduce neuroinflammation and protect the injured spinal cord neurons. **(A)** Fluorescently stained images showing CCR7^+^ (green)/CD68^+^ (red) M1 macrophage cells in three groups (low magnification and high magnification). **(B)** Bar charts show the ratio of CCR7^+^/CD68^+^ cells to CD68^+^ cells in the injury/graft site of the spinal cor. **(C)** Fluorescently stained images showing CD206^+^ (green)/CD68^+^ (red) M2 macrophage cells in three groups (low magnification and high magnification). **(D)** Bar charts show the ratio of CD206^+^/CD68^+^ cells to CD68^+^ cells in the injury/graft site of the spinal cord. **(E)** Western blot analysis of protein expression (CCR7, CD206, CD68). **(F)** Quantitative analysis of Western blot. **(G–H)**: ELISA was performed to detect the concentrations of IL-10 and TNF-α from the injury/graft site of the spinal cord. **(I)** Nissl staining showed the neurons in Clark’s nuclei of the L1. **(J)** Bar charts showing the number of neurons. Statistic difference: *p < 0.05; **p < 0.01.

Macrophages/microglia orchestrate neuroinflammation after SCI. Based on their function, macrophages/microglia are classified into the pro-inflammatory M1 and anti-inflammatory M2 phenotypes (Sica and Mantovani, 2012). To investigate how sEVs uptake may change the phenotype and function of macrophages/microglia in the injured/injection site of the spinal cord, CCR7, a marker of pro-inflammatory M1 macrophages, or CD206, a marker of anti-inflammatory M2 macrophages were used to identify the subtypes of the CD68^+^ cells. The immuno-histochemistry results showed that an increased portion of CD206^+^/CD68^+^ M2 macro-phages and a decreased portion of CCR7^+^/CD68^+^ M1 macrophages were observed in the injury/injection site of the spinal cord in the 4D-sEVs group relative to the SCI and 2D-sEVs groups (Figures 3A–D). Similarly, western blot results showed that the protein expressions of CCR7 and CD68 were significantly decreased, and that of CD206 was increased in the injury/injection site of the spinal cord in the 4D-sEVs group, relative to the SCI and 2D groups (Figures 3E,F). ELISA results showed that the expression of anti-inflammatory factors, IL-10, was significantly increased, and the expression of pro-inflammatory factors, TNF-α, was significantly decreased in the injury/injection site of the spinal cord in the 4D-sEVs group relative to the SCI and 2D-sEVs groups (Figures 3G,H). These results suggested that sEVs, especially 4D-sEVs, could po-larize macrophages/microglia from the M1 toward the M2 phenotype in the injury/injection site of the spinal cord.

To detect the neuroprotective effect of sEVs administration, the survival of the dorsal nucleus (Clark’s neurons) of the lumbar spinal cord segment 1 (L1) was selected for assessment because these large diameter neurons are morphologically readily identifiable, undergo degeneration approximately 1 month after a low thoracic lesion, and are sensitive to the treatments (McBride et al., 1988; Shibayama et al., 1998). Nissl staining showed that compared with normal spinal cord, neurons in the dorsal nucleus undergo massive apoptosis after crush injury. However, the number of surviving Clark’s neurons at L1 4 weeks after SCI was notably increased in the 4D-sEVs group relative to the 2D-sEVs and SCI groups (Figures 3I,J). These results suggested that although both 4D-sEVs and 2D-sEVs have inhibitory effects on inflammation, 4D-sEVs is more effective than 2D-sEVs in attenuating the inflammatory response in the injured injection site by inducing M2 polarization and, therefore, help the survival of spinal neurons after SCI.

### 3.4 Four dimensional culture-derived sEVs induced LPS-activated RAW264.7 polarization from the M1 to the M2 phenotype

**FIGURE 4:**
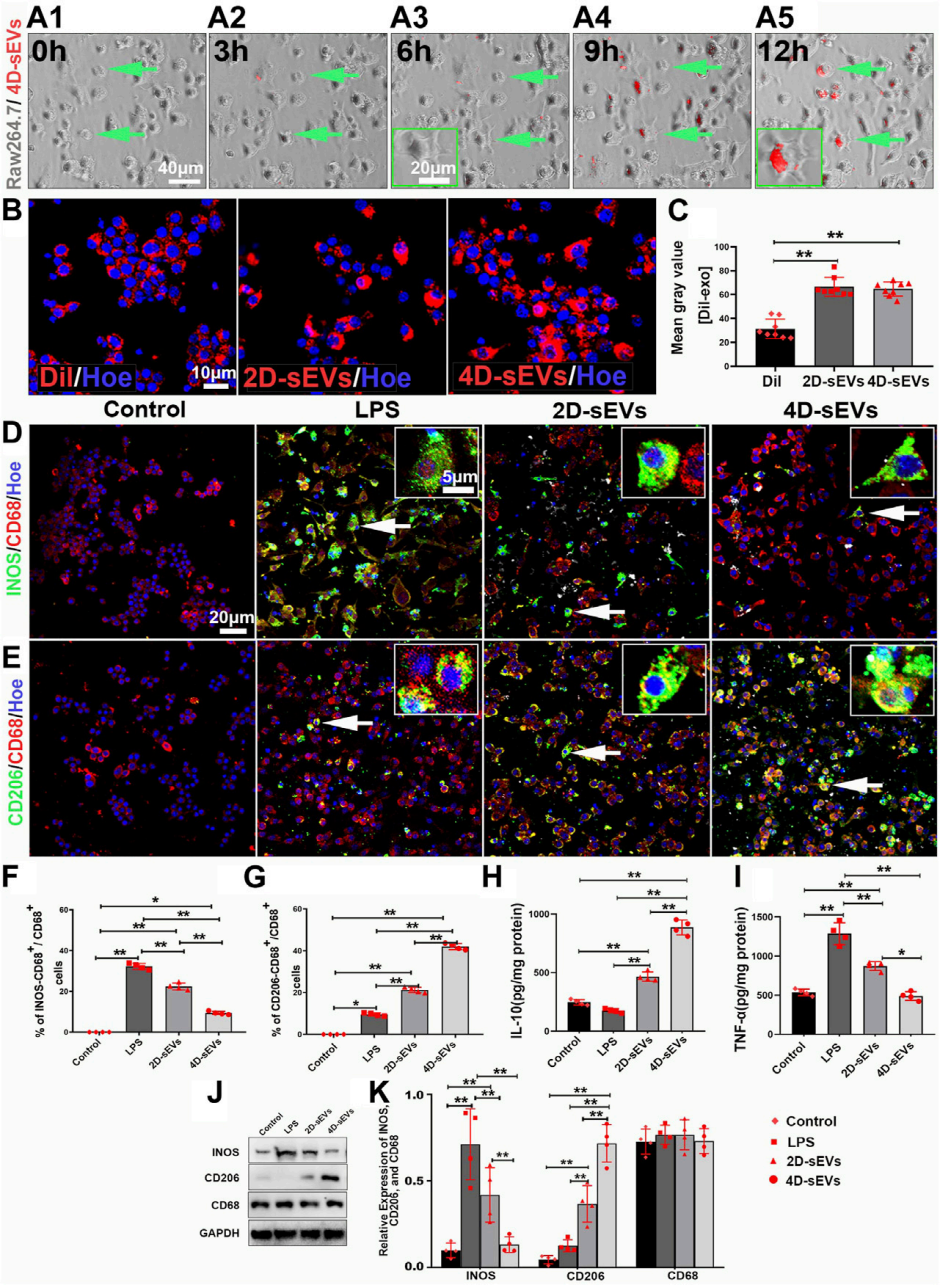
Four dimensional-derived sEVs promote LPS pre-induced activation of M1 to M2 type macrophage polarization *in vitro*. A1–A5: Live-cell imaging system to observe the uptake of Dil-labeled sEVs in RAW264.7. **(B)** The fluorescently stained images show that RAW264.7 cells uptake Dil dye and Dil-labeled sEVs. **(C)** Bar charts showing the mean fluorescence intensity *in vitro*. **(D–E)**: Fluorescently stained images showing INOS^+^ (green)/CD68^+^ (red) M1 macrophage cells and CD206^+^ (green)/CD68^+^ (red) M2 macrophage cells in the three groups, Control (non-LPS-induced) RAW264.7 cells do not express INOS and CD206. **(F)** Bar charts showing that the ratio of INOS^+^/CD68^+^ cells to CD68^+^ cells. **(G)** Bar charts showing that the ratio of CD206^+^/CD68^+^ cells to CD68^+^ cells. **(H, I)**: ELISA was performed to detect the concentrations of IL-10, and TNF-α from the supernatant of RAW264.7 cells. **(J)** Western blot analysis of protein expression (INOS, CD206, CD68) *in vitro*. **(K)** Quantitative analysis of Western blot. Statistic difference: *p < 0.05; **p < 0.01.

To explore how 4D-sEVs induced macrophage/microglia polarization from the M1 to the M2 phenotype, the macrophage cell line RAW264.7 was studied *in vitro*. RAW264.7 cells were activated by LPS and analyzed by adding sEVs from different groups. Red fluorescence was seen in the cytoplasm of RAW264.7 cells after adding DiI-labeled 4D sEVs after 3 h and lasted for at least 12 h (Figures 4A1–A5). After the Dil^+^ dye or sEVs were engulfed by RAW264.7 cells, the fluorescence intensity in RAW264.7 cells were significantly increased in the 2D-sEVs and 4D-sEVs groups relative to the Dil group. However, the fluorescence intensity in RAW264.7 cells showed no difference between the 2D-sEVs and 4D-sEVs groups (Figures 4B,C). Quantification of DiI-labeled RAW264.7 cells showed that 87.98% ± 5.27% of cells in the 2D-sEVs group and 91.00 %± 4.97% in the 4D-sEVs group had engulfed DiI-labeled sEVs, but there was no statistical difference in the amount of sEVs uptake between these two groups.

Lipopolysaccharide (LPS) effectively activates RAW264.7 cells towards the M1 phenotype. There was neither INOS (M1 phenotype) nor CD206 (M2 phenotype) expression in RAW264.7 cells without LPS stimulation (the Control group). The INOS expression was detected in RAW264.7 cells soon after LPS stimulation (the LPS group), showing an M1 phenotype polarization (INOS^+^/CD68^+^ (31.90% ± 2.36%) vs CD206^+^/CD68^+^ (8.36% ± 2.03%). However, after sEVs treatment, the M1 phenotype was suppressed despite LPS stimulation (INOS^+^/CD68^+^ (19.75% ± 8.39%) vs CD206^+^/CD68^+^ (18.27% ± 2.03%)) in the 2D-sEVs group and (INOS^+^/CD68^+^ (5.29% ± 2.02%) vs CD206^+^/CD68^+^ (41.12% ± 5.95%)) in the 4D-sEVs group. M2 polarization was more significant in the 4D-sEVs group compared with the 2D group (Figures 4D,E). ELISA results showed that the expression of anti-inflammatory factors, IL-10, was increased, and that of pro-inflammatory factors, TNF-α, were decreased in the 4D-sEVs group relative to the LPS and 2D groups (Figures 4H,I).Western blotting confirmed a similar trend of INOS and CD206 protein expression in the LPS, the 2D-sEVs, and the 4D-sEVs groups (Figures 4J,K). These results suggest that sEVs, especially 4D-sEVs, can induce macrophage polarization from the M1 to the M2 phenotype, with altered secretomes favoring anti-inflammation.

### 3.5 Upregulation of IGFBP2/EGFR protein in four dimensional culture-derived sEVs

**FIGURE 5:**
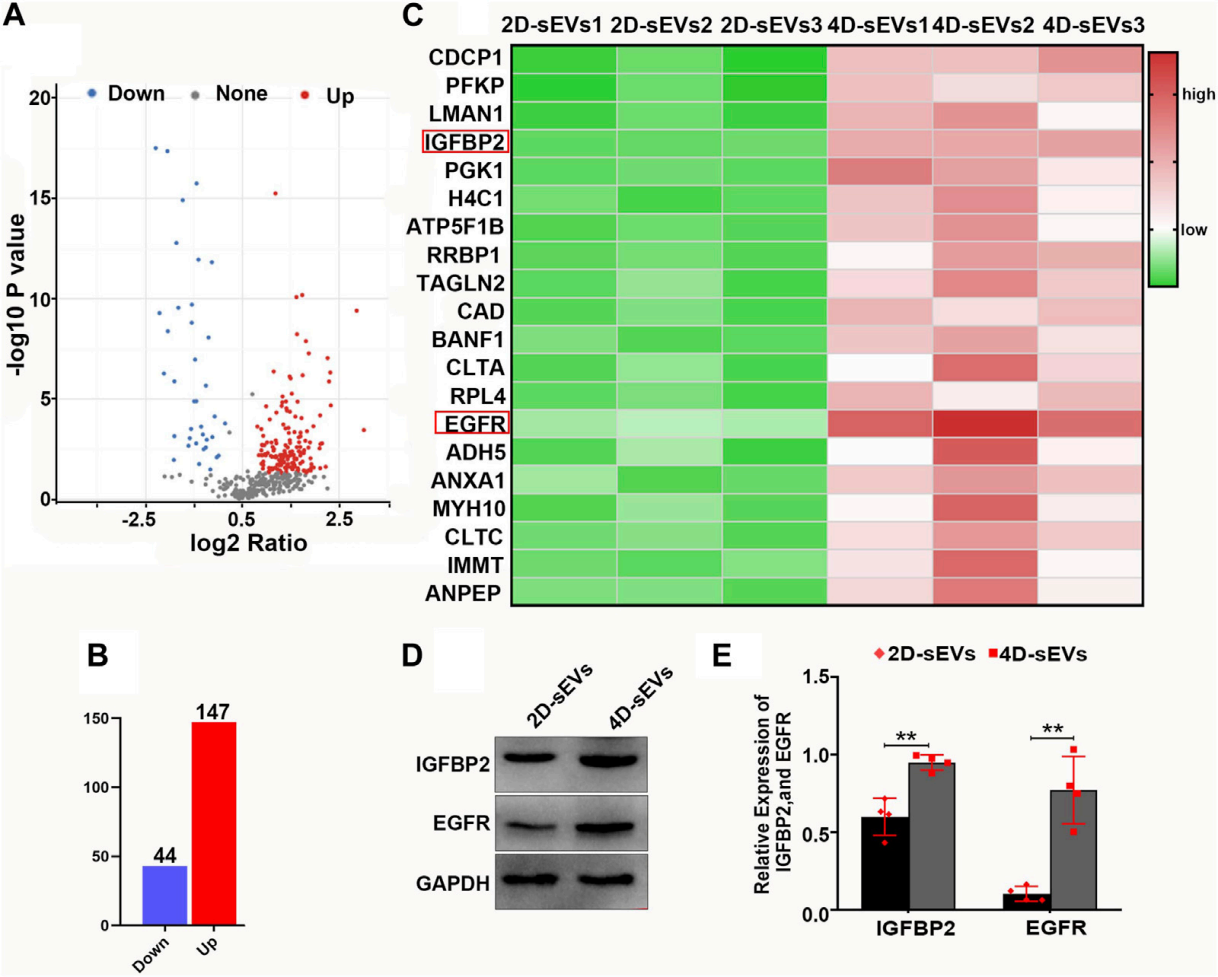
Proteomic analysis of sEVs and comparison of the results for both 2D- and 4D-sEVs. **(A, B)**: Protein changes detected in the 2D- and 4D-sEVs groups are summarized on a volcano plot and histogram. **(C)**: Heat map showing the top 20 proteins with the most variation in the 2D- and 4D-sEVs groups. IGFBP2/EGFR is associated with M1 to M2 polarization of macrophages. **(D)**: The expressions of IGFBP2 and EGFR in two groups of sEVs were tested by western blotting. **(E)**: Quantitative analysis of Western blot. Statistic difference: **p < 0.01.

To investigate the different biological effects between 2D-sEVs and 4D-sEVs, proteomics was used to identify the protein profile in the 2D-sEVs and 4D-sEVs groups. The results showed that compared with 2D-sEVs, 147 proteins were increased, and 44 proteins were decreased in 4D-sEVs (Figures 5A,B). Twenty of the most upregulated proteins from the total 147 proteins were selected and analyzed. Among them, IGFBP2/EGFR, which is reported to be involved in microglia polarization (Chua et al., 2016; Li et al., 2020; Sun et al., 2021), was studied. A proteomics study showed that the amount of IGFBP2/EGFR protein was significantly different between the 2D-sEVs and 4D-sEVs groups (Figure 5C). Western blotting further confirmed that the amount of IGFBP2/EGFR protein was higher in the 4D-sEVs compared with the 2D-sEVs group (Figures 5D,E).

### 3.6 Four dimensional culture-derived sEVs-transported IGFBP2/EGFR protein activates the STAT3 pathway in RAW264.7 macrophages

**FIGURE 6:**
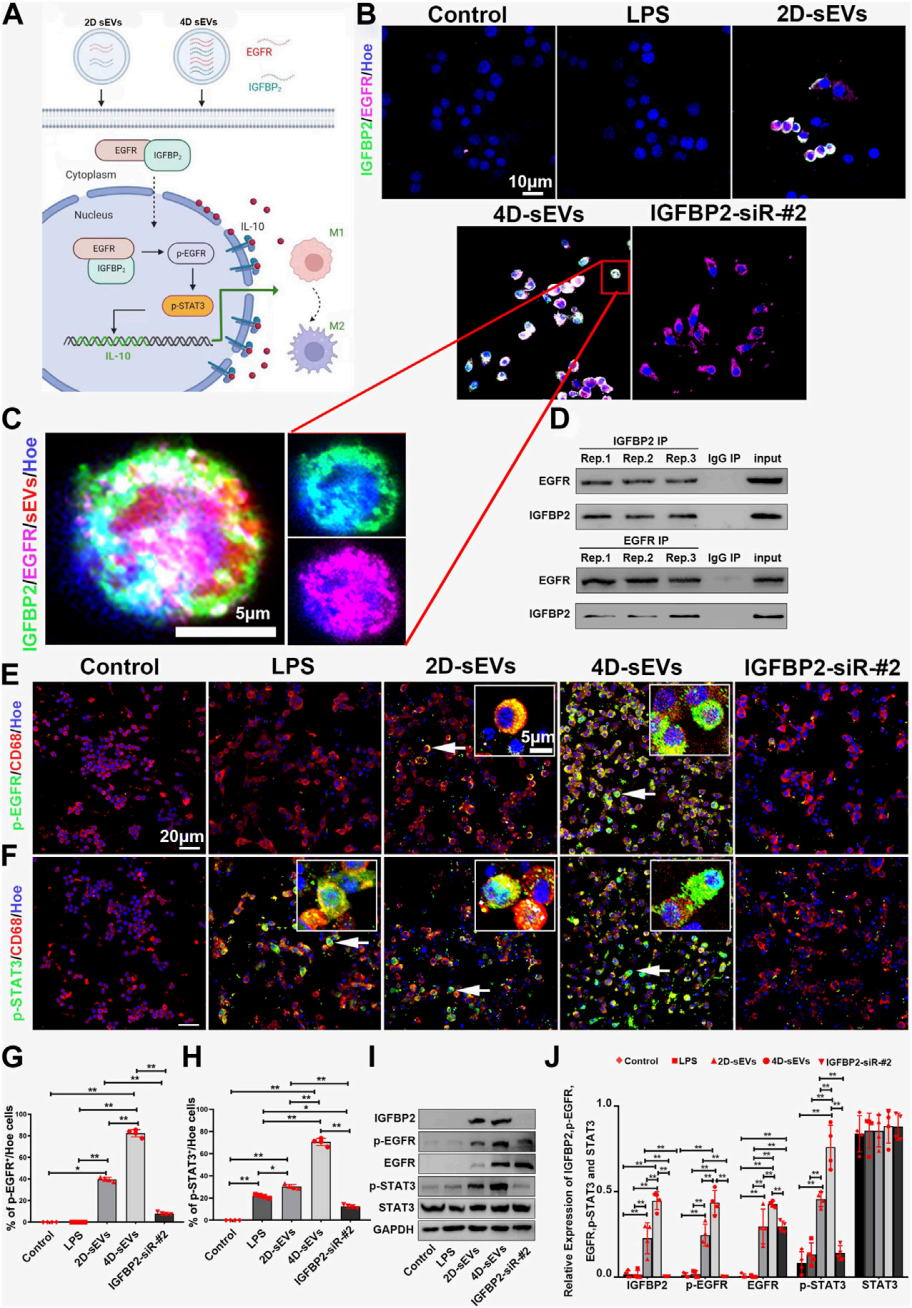
The IGFBP2/EGFR protein transported within sEVs activates the STAT3 pathway in RAW264.7 cells *in vitro*. **(A)** Diagram showing how sEVs-transported IGFBP2/EGFR protein promotes M1 to M2 polarization of macrophages. **(B)** Fluorescently stained images showing co-labeled IGFBP2 (green) and EGFR (purple) in RAW264.7 cells within each group. **(C)** The high magnification of B in the frame. **(D)** Co-immunoprecipitation (IP) of IGFBP2 and EGFR in RAW264.7 cells. **(E)** Fluorescently stained images showing p-EGFR^+^/CD68^+^cells in each group. **(F)** Fluorescently stained images showing p-STAT3^+^/CD68^+^cells in each group. **(G, H)**: Bar charts showing p-EGFR and p-STAT3 activation rate. **(I)** Western blot analysis of protein expression (IGFBP2, p-EGFR, EGFR, p-STAT3, STAT3). **(J)** Quantitative analysis of Western blot. Statistic difference: *p < 0.05; **p < 0.01.

The presumed signaling pathway of the IGFBP2/EGFR protein regulating the M1 to M2 phenotype polarization is illustrated by the schematic diagram (Figure 6A). Immunofluorescence staining suggested that nearly all RAW264.7 cells had an intracellular expression of IGFBP2 and EGFR protein after 4D-sEVs treatment. However, none or few of them had IGFBP2 and EGFR protein expression of either after receiving the DiI dye (the Control group), LPS stimulation (the LPS group), or 2D-sEVs group (Figures 6B,G). To further explore the function of IGFBP2 in regulating EGFP/STAT3-dependent macrophage polarization, siRNA targeting IGFBP2 was used to downregulate IGFBP2 expression in MSC 3D cultures (Supplementary Figure S2A–H). RAW264.7 cells that received 4D-sEVs pretreated with IGFBP2-siRNA (the IGFBP2-siR-#2 group) demonstrated scarce IGFBP2 expression but no change in EGFR expression (Figure 6B). The intracellular binding of IGFBP2 and EGFR was confirmed by Co-Immunoprecipitation (Co-IP) of both proteins extracted from RAW264.7 cells receiving 4D-sEVs (Figure 6D).

Immunofluorescence results showed that RAW264.7 cells had an increased intracellular expression of p-EGFR after engulfing 2D-sEVs or 4D-sEVs. However, none or few of them demonstrated p-EGFR expression after receiving DiI dye (the Control group), LPS stimulation (the LPS group), 2D-sEVs, or 4D-sEVs with IGFPB2-siRNA pretreatment (the IGFBP2-siR-#2 group) (Figure 6E). The expression of p-STAT3 was more evident in the 4D-sEVs group, the 2D-sEVs group, and the LPS group compared with the Control group and the IGFBP2-siR-#2 group (Figure 6F). The fluorescence quantification showed that p-EGFR and p-STAT3 immunosignal more were actived in the 4D-sEVs group relative to the other groups (Figures 6G,H). Similarly, western blotting validated the significant upregulation of IGFBP2, EGFR, p-EGFR, and p-STAT3 in RAW264.7 cells in the 4D-sEVs group compared with other groups. Notably, upregulation of IGFBP2, EGFR, p-EGFR, and p-STAT3 was compromised when IGFBP2 in 4D-sEVs had been downregulated by siRNA pretreatment (Figures 6I,J). In addition, when we inhibited STAT3 phosphorylation with S3I-201(NSC74859), ELISA detected significant downregulation of IL10 secretion (Supplementary Figures S2I–2K). The above data shows that, culture-derived sEVs-transported IGFBP2/EGFR protein activates the phosphorylation of STAT3, promoting the secretion of IL10.

### 3.7 Downregulation of IGFBP2 attenuated M2 microglia polarization induced by four dimensional culture-derived sEVs

**FIGURE 7:**
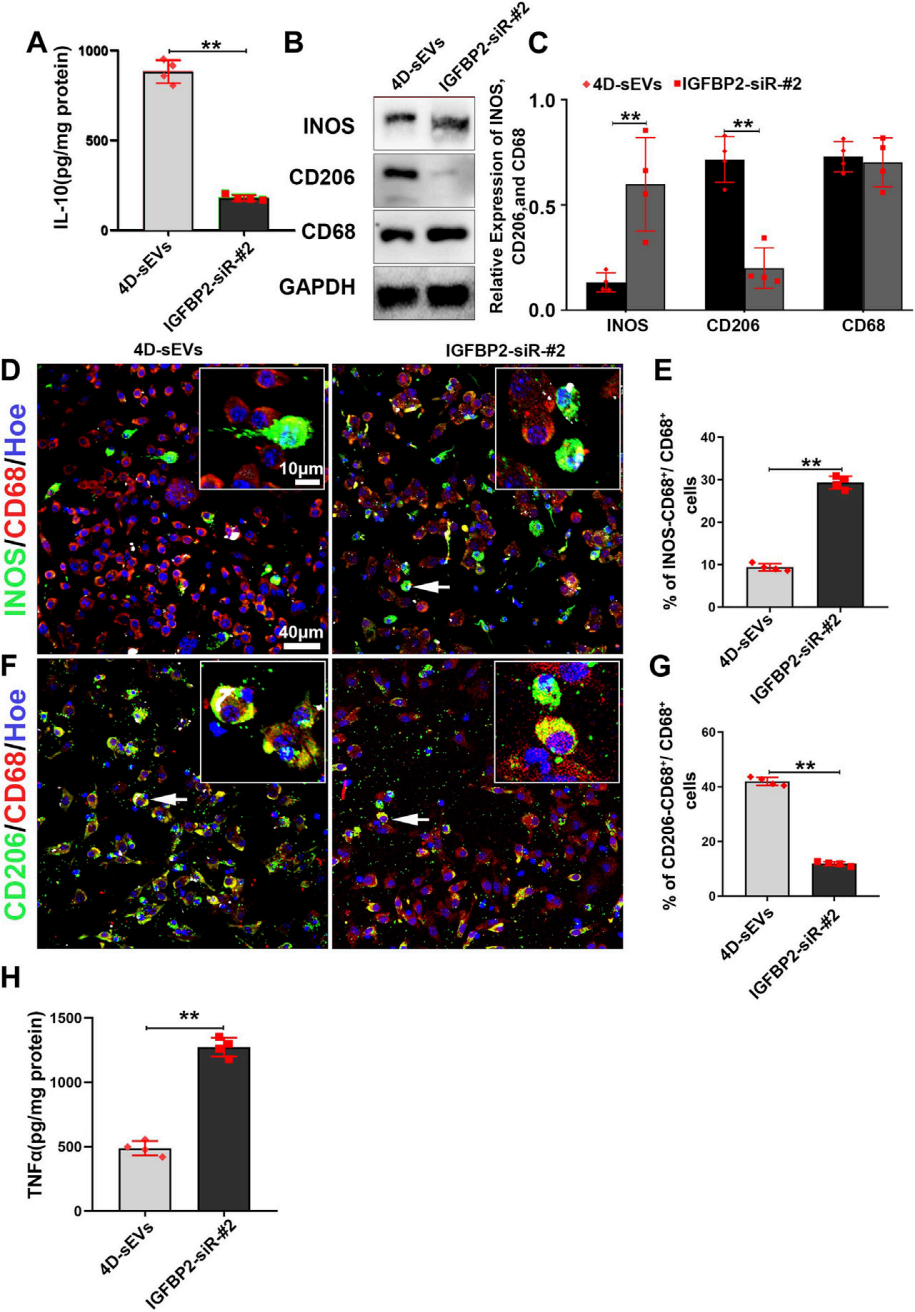
Interfering with the IGFBP2 protein expression inhibits M1 to M2 polarization of macrophages *in vitro*. **(A)** ELISA was performed to detect the concentrations of IL-10 from the supernatant of RAW264.7 cells. **(B)** Western blot analysis of protein expression (INOS, CD206, CD68). **(C)** Quantitative analysis of Western blot. **(D)** Fluorescently stained images showing INOS^+^ (green)/CD68^+^ (red) M1 macrophage cells in two groups. **(E)** Bar charts showing that the ratio of INOS^+^/CD68^+^ cells to CD68^+^ cells. **(F)** Fluorescently stained images showing CD206^+^ (green)/CD68^+^ (red) M2 macrophage cells in two groups. **(G)** Bar charts showing that the ratio of CD206^+^/CD68^+^ cells to CD68^+^ cells. **(H)** ELISA was performed to detect the concentrations of TNF-α from the supernatant of RAW264.7 cells. Statistic difference: **p < 0.01.

Downstream of EGFR/STAT3, the expression of Interleukin-10 (IL-10) is critical for the M1 to M2 polarization of the activated macrophage. As a M2 dominantly expressed cytokine, IL-10 has been shown to inhibits nitric oxide formation by decreasing INOS protein expression in macrophage (Cunha et al., 1992; Ogando et al., 2004). The secretion of IL-10 in the IGFBP2-siR-#2 group was significantly decreased compared with the 4D-sEVs group (Figure 7A). As a result, the expression of INOS was significantly increased, and CD206 decreased in the IGFBP2-siR-#2 group compared with the 4D-sEVs group (Figures 7B,C). Immunofluorescence results showed that INOS^+^ M1 and CD206^+^ M2 macrophages were observed in both the IGFBP2-siR-#2 and 4D-sEVs groups (Figures 7D,F). However, the positive rate of INOS^+^ cells was significantly increased, and CD206^+^ cells were significantly decreased in the IGFBP2-siR-#2 group compared with 4D-sEVs (Figures 7E,G). ELISA results showed that the expression of pro-inflammatory factors, TNF-α, was significantly increased in the IGFBP2-siR-#2 group than 4D-sEVs group (Figure 7H). These results suggest an instrumental role for IGFBP2 in 4D-sEVs induced M1 to M2 polarization.

### 3.8 Four dimensional culture-derived sEVs induced M2 polarization in the injury/injection site of the spinal cord via IGFBP2/EGFR pathway

**FIGURE 8:**
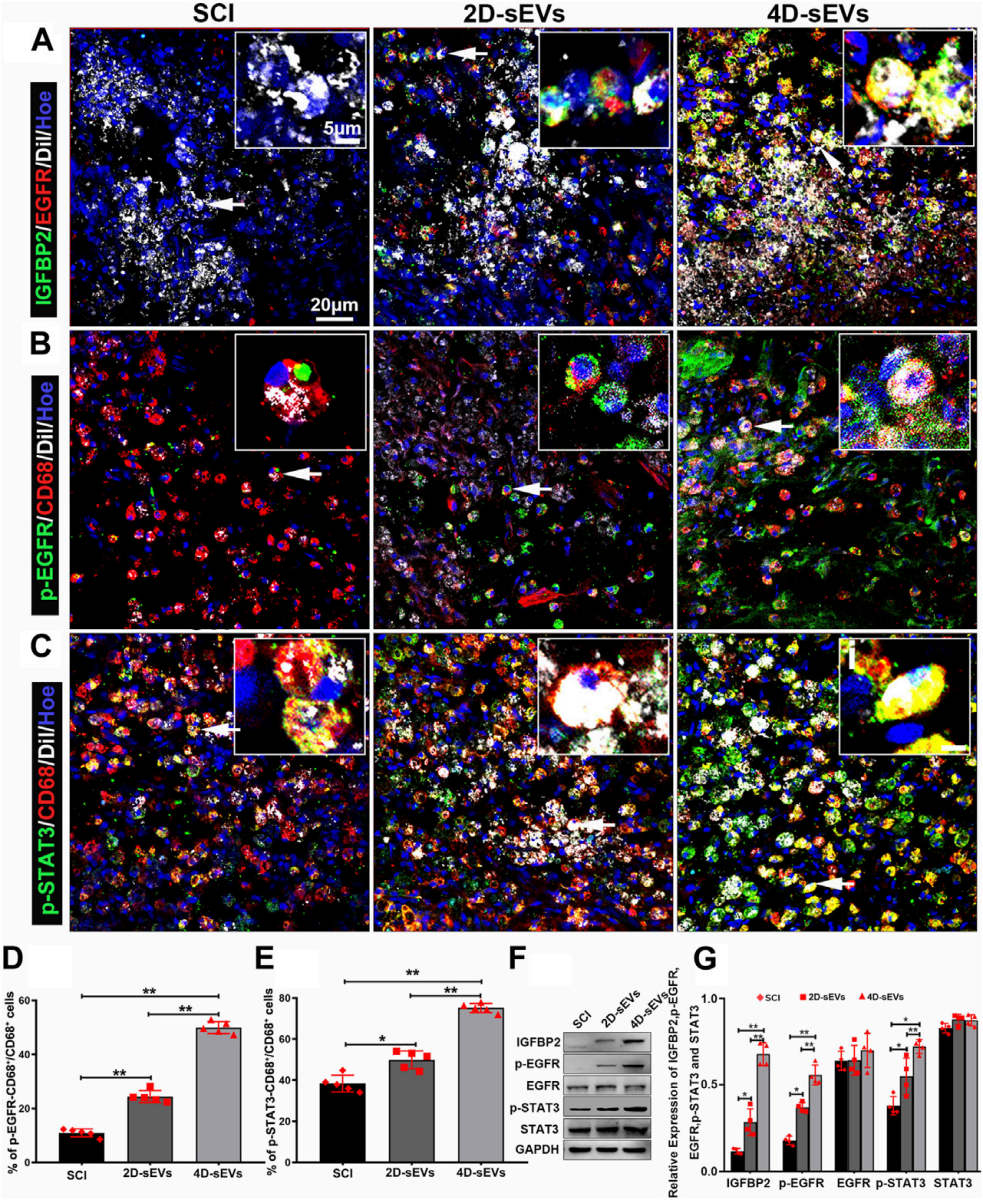
SEVs-transported IGFBP2/EGFR protein activates the STAT3 pathway in the injury/graft site of the spinal cord. **(A)** Fluorescently stained images showing co-labeled IGFBP2 (green) and EGFR (red) in cells taking up sEVs (white) in the injury/graft site of the spinal cord in three groups. **(B)** Fluorescently stained images showing p-EGFR^+^/CD68^+^ cells to CD68 cells in the injury/graft site of the spinal cord. **(C)** Fluorescently stained images showing p-STAT3^+^/CD68^+^ cells to CD68 cells in the injury/graft site of the spinal cord. **(D)**, **(E)** Bar charts showing the fluorescence intensity of p-EGFR and p-STAT3. **(F)** Western blot analysis of protein expression (IGFBP2, p-EGFR, EGFR, p-STAT3, STAT3). **(G)**: Quantitative analysis of Western blot. Statistic difference: *p < 0.05; **p < 0.01.

To determine whether M2 microglial polarization at injury/injection sites *in vivo* is mediated by IGFBP2/EGFR protein signaling, we performed immunofluorescence assays. The results showed that IGFBP2/EGFR protein had been observed in the cells that engulfed sEVs (Figure 8A), and p-EGFR and p-STAT3 have been observed in CD68^+^ macrophages/activated microglia (Figures 8B,C). The fluorescence quantification showed that p-EGFR and p-STAT3 were expressed in the 4D-sEVs group and were statistically significantly different from 2D-sEVs and SCI groups (Figures 8D,E). Similarly, more p-EGFR and p-STAT3 were expressed in the 4D-sEVs group and validated by western blot (Figures 8F,G). These results suggested that after macrophages engulfed sEVs in the injury site of the spinal cord, IGFBP2/EGFR proteins aggregated in the macrophages and activated the STAT3 signaling pathway, which induced polarization of macrophage/microglia from M1 toward the M2 phenotype.

## 4 Discussion

Obtaining disease-targeting sEVs with promising efficacy remains a challenging task. Even though there has been positive feedback after their application in several diseases (Guo et al., 2019; Jiang et al., 2019; Zhang and Yu, 2019), dynamic pathology changes may require corresponding therapeutic elements. In this study, we have, for the first time, reported the characteristics and therapeutic poten-tials of the sEVs derived from the 4D culture of MSCs (4D-sEVs). Rather than simply seeding cells in a 3D scaffold, the 4D culture requires a prolonged culture time and sufficient interactions between the seeded cells and the ambient microenvironment, leading to a specific niche favorable to the resident MSCs with notable alterations in the sEVs pattern. Indeed, the results of this study have shown that 4D-sEVs were not only larger in number and size but also enveloped somewhat different proteins relative to the sEVs from 2D culture. Further analysis using proteomics revealed that 147 proteins were increased and 43 proteins were decreased in four dimensional culture-derived sEVs compared with 2D sEVs. This change in protein profile enabled 4D-sEVs to have a notable elevation in anti-neuroinflammation capability suitable for repairing trauma in the central nervous system (CNS), such as SCI. Although the exact mechanism is still largely unknown, it was noted in this study that the mutual binding of upregulated EGFR and IGFBP2 and their nuclear internalization might be essential for STAT3-dependent M1 to M2 polarization of the activated macrophages/microglia, the main contributors to neuroinflammation after SCI. Given the comprehensive development of sEVs as drug delivery carriers (Familtseva et al., 2019; Zhao et al., 2020; Liang et al., 2021), the 4D culture technique may yield qualitative and quantitative changes for sEVs-based drugs, allowing targeted treatment for complicated diseases such as SCI (Figure 8N).

The 3D culture technique in our previous studies and the current one highlighted the “time” factor during the 3D culture process, a principle of “4D” biology (Tibbitt and Anseth, 2012). A homeostatic niche is expected to be achieved in such a system after the dynamic interactions between the seeded cells and the surrounding microenvironment, including the scaffold and the neighboring cells (Fuchs and Blau, 2020; McKinley et al., 2020). Although there is a small number of studies applied a prolong culture time to generate exsomes from 3D cultured MSCs(Li et al., 2019), the detailed mechanism regarding the biology and therapeutic potentials of 4D-sEVs is largely still unknown, it is plausible that intercellular communication via the sEVs in 4D environment would be different from that in 2D culture. Indeed, in the current study, the number, size, and inner protein contents of the sEVs exhibited significant differences between the 4D-sEVs and 2D-sEVs groups. It is hypothesized that the outside matrix dynamically integrating with the scaffold may finally lead to a stable microenvironment, allowing for the survival of cells in the 4D environment (Lai et al., 2013; Zeng et al., 2015; Lai et al., 2018; Wu et al., 2018; Lai et al., 2019; Li et al., 2021). In this study, enriched IGFBP2 in 4D-sEVs has been screened out by proteomics. It has been shown that IGFBP2 supports cell survival (Huynh et al., 2011; Khan, 2019; Shen et al., 2019). Therefore, it could be inferred that sEVs, as the intercellular communication mediator, may transmit trophic signals to support various cell types. Given the drastic influence that the microenvironment imposes on the MSC function (Engler et al., 2006; Mohyeldin et al., 2010; Chaudhuri et al., 2020), applying the 4D culture methodology may help generate sEVs with a new function to ameliorate the unfavorable factors in the disease microenvironment (Wechsler et al., 2021). Therefore this regimen may help in the targeted design of sEVs products to treat dif-ferent disease conditions.

The secondary injury cascades following the initial SCI result in a hostile microenvironment dominated by unrelieved neuroinflammation, which hinders the natural healing of the injured spinal cord. Studies have validated the anti-inflammation effect of the transplanted MSCs in the injured spinal cord (Guo et al., 2019; Pang et al., 2021). Among all the mechanisms, intercellular communication via extracellular vesicles may be a key factor for MSCs in reducing neuroinflammation (Huang et al., 2017; Zhang et al., 2018). This study first identified (by proteomics analysis) the upregulation of IGFBP2/EGFR proteins in 4D-sEVs during inflammation modulation. After phagocytosis by macrophages/microglia, IGFBP2/EGFR within the sEVs induced downstream STAT3 phosphorylation, which proved to be the key molecular event during M1 to M2 polarization (Chua et al., 2016; Li et al., 2020; Sun et al., 2021), leading to increased IL-10 expression. IL-10 facilitated M2 polarization through paracrine or autocrine mechanisms (Makita et al., 2015; Jung et al., 2017; Mahon et al., 2020), elevating the macrophage/microglia subpopulation that was anti-inflammatory. Indeed, compared with 2D-sEVs, 4D-sEVs exhibited a stronger immunoregulatory effect on the SCI microenvironment, which is expected to restore the SCI structurally and functionally (David and Kroner, 2011). As a result, the surviving neurons in L1 Clark’s nucleus, which project to the cerebellum and are sensitive to the insults at the T10 level (Schmitt et al., 1999), were significantly improved after 4D-sEVs treatment compared with 2D-sEVs treatment or the injury control, suggesting a neuroprotective microenvironment endowed by 4D-sEVs. Therefore, the anti-inflammation feature of 4D-sEVs may have great potential for regulating inflammatory responses after a variety of injuries. However, since the biological contents within 4D-sEVs are complicated, whether and how other molecules besides IGFBP2/EGFR proteins may help SCI structural and functional repair remain to be studied. With better understanding of the 4D biology following a prolonged culture time of MSCs in the 3D scaffold, the efficacy of 4D-sEVs for more comprehensive application to other diseases warrants further investigation.

## 5 Conclusion

4D culture technology generated more sEVs with larger sizes and varied proteo-mic profiles. The sEVs contained upregulated IGFBP2/EGFR proteins, which could po-larize macrophages/microglia from the pro-inflammatory M1 to pro-regenerative M2 phenotype via the EGFR/STAT3 pathway. This resulted in reduced neuroinflammation and neuronal loss at the spinal cord injury site. Therefore, the MSC 4D culture-derived sEVs technology could be developed into an effective treatment for SCI.

## Data Availability

The original contributions presented in the study are publicly available, further inquiries can be directed to the corresponding author. The accession number of proteomics raw data is PXD040516.
