# Supplementary material for: Small extracellular vesicles derived from four dimensional-culture of mesenchymal stem cells induce alternatively activated macrophages by upregulating IGFBP2/EGFR to attenuate inflammation in the spinal cord injury of rats
Source: Front Bioeng Biotechnol. 2023 Apr 28;11:1146981. doi: 10.3389/fbioe.2023.1146981 (PMC10176095; doi:10.3389/fbioe.2023.1146981)
Supplement: Supplementary file 3 [file DataSheet1.docx]

Supplementary Material


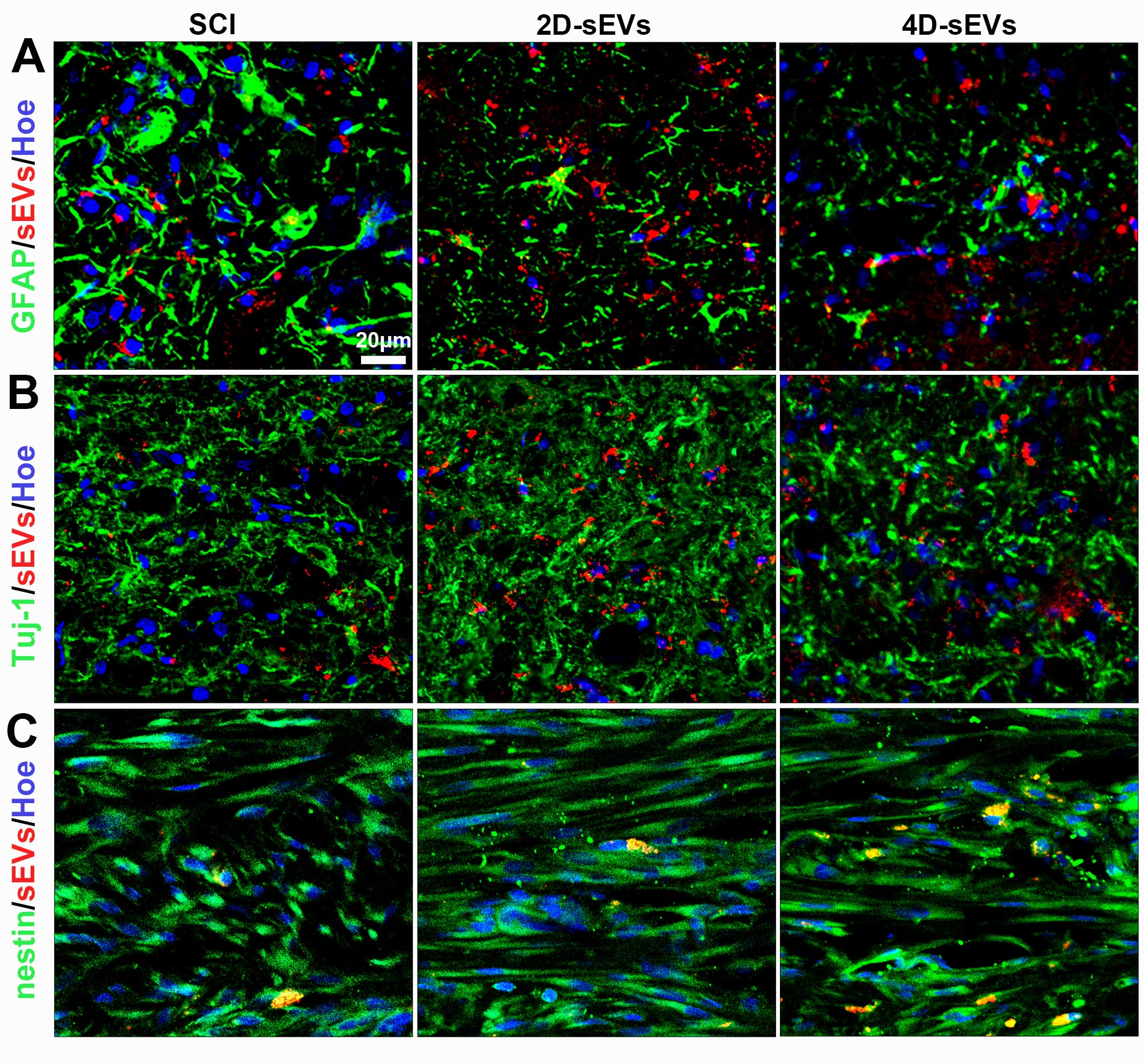


**Figure Supplement 1. The phagocytosis of sEVs by cells in the spinal cord injury site.** A–C: The fluorescently stained images show GFAP^+^ cell, Tuj-1^+^ cell, nestin^+^ cell uptake of Dil and Dil-sEVs in the injury/graft site of the spinal cord.


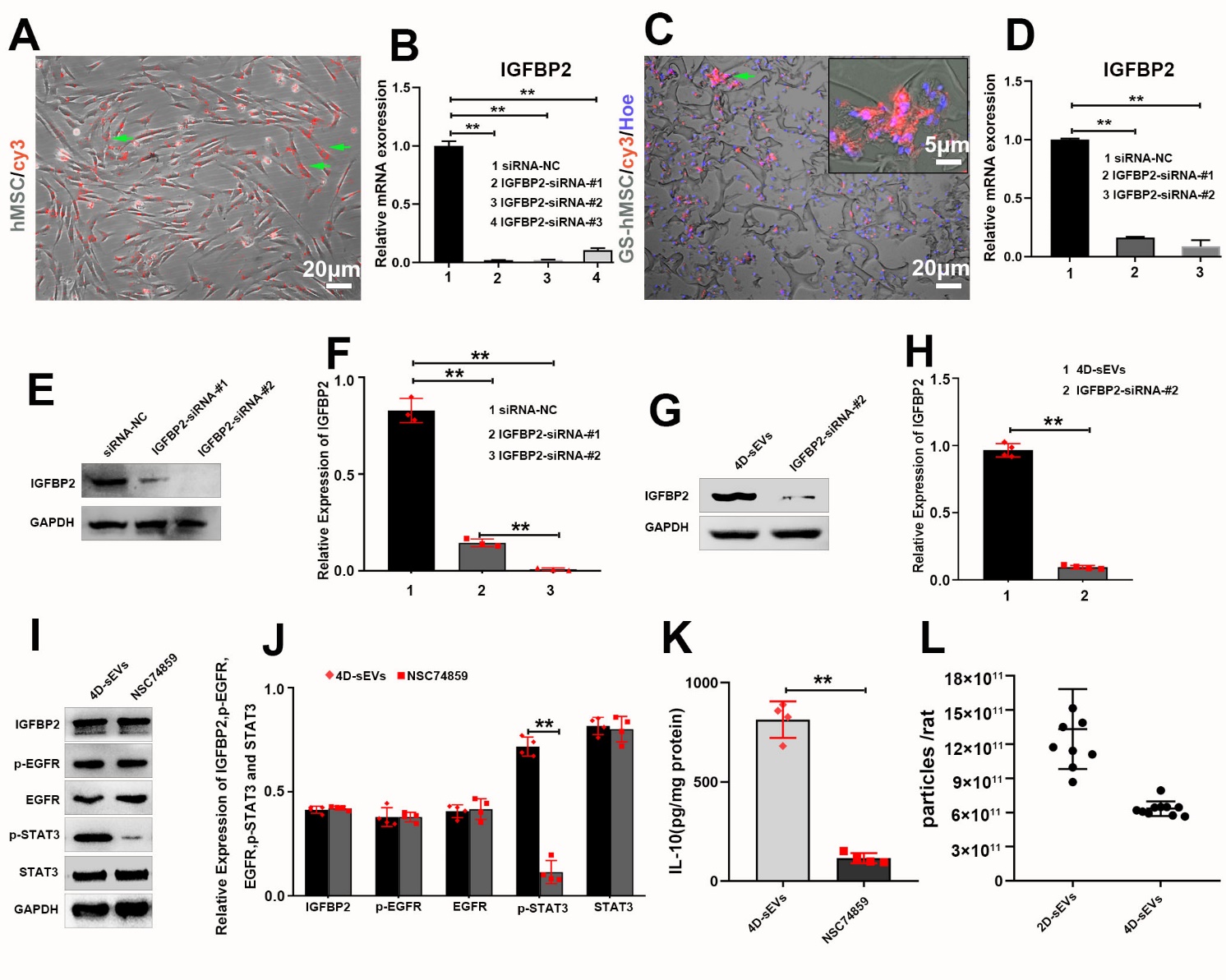


**Figure Supplement 2. IGFBP2-siR-#2 can effectively interfere with IGFBP2 expression of hUC-MSC-derived sEVs**. A: Cy3-labeled null RNA can be observed in almost all hUC-MSCs under a fluorescence microscope. B: PCR detection of IGFBP2 mRNA expression of different interfering sequences in hUC-MSCs. C: Cy3-labeled null RNA can be observed in almost all tissueoid culture of MSCs under a fluorescence microscope. D: PCR detection of IGFBP2 mRNA expression in the tissueoid culture of MSCs. E: The expressions of IGFBP2 were determined in the tissueoid culture of MSCs by Western blot. F: Quantitative analysis of Western blot. G: The expressions of IGFBP2 in tissueoid culture-derived sEVs were tested by Western blot. H: Quantitative analysis of Western blot. I: Western blot analysis of protein expression (IGFBP2, p-EGFR, EGFR, p-STAT3, STAT3) in 4D-sEVs and NSC74859 groups. J: Quantitative analysis of Western blot. K: ELISA was performed to detect the concentrations of IL-10 from the supernatant of RAW264.7 cells in 4D-sEVs and NSC74859 groups.L: Injected with number of EVs corresponding to 500ug protein for each rat. Statistic difference: **p < 0.01.

**Supplementary Table 1**

Primary and secondary antibodies.

| Antibodies | Species | Type | Dilution | Source |
| --- | --- | --- | --- | --- |
| CD68 | Mouse | Monoclonal IgG | 1:200/1:1000 | Sigma, St. Louis, USA |
| CD68  CCR7  CD206  INOS  IGFBP2  EGFR  EGFR  p-EGFR  STAT3  p-STAT3  GAPDH | Rabbit  Rabbit  Rabbit  Rabbit  Rabbit  Rabbit  Mouse  Rabbit  Mouse  Rabbit  Rabbit | Monoclonal IgG  Monoclonal IgG  Polyclonal IgG  Monoclonal IgG  Monoclonal IgG  Monoclonal IgG  Monoclonal IgG  Monoclonal IgG  Monoclonal IgG  Monoclonal IgG  Polyclonal IgG | 1:200  1:500/1:1000  1:200/1:1000  1:200/1:1000  1:30/1:1000  1:100/1:1000  1:200/1:1000  1:200/1:1000  1:200/1:1000  1:200/1:1000  1:1000 | Abcam, London, UK  Abcam, London, UK  Abcam, London, UK  Abcam, London, UK  Cell Signaling Technology, Danvers, USA  Cell Signaling Technology, Danvers, USA  Abcam, London, UK  Cell Signaling Technology, Danvers, USA  Cell Signaling Technology, Danvers, USA  Cell Signaling Technology, Danvers, USA  Sigma, St. Louis, USA |
| Alexa 488 conjugated anti rabbit secondary antibody | Goat | Polyclonal IgG | 1:1000 | Abcam, London, UK |
| Alexa 647 conjugated anti mouse secondary antibody | Goat | Polyclonal IgG | 1:1000 | Abcam, London, UK |
| HRP-conjugated anti mouse secondary antibody  HRP-conjugated anti Rabbit secondary antibody | Goat  Goat | Polyclonal IgG  Polyclonal IgG | 1:2000  1:5000 | Jackson ImmunoResearch, West Grove, USA  Jackson ImmunoResearch, West Grove, USA |

**
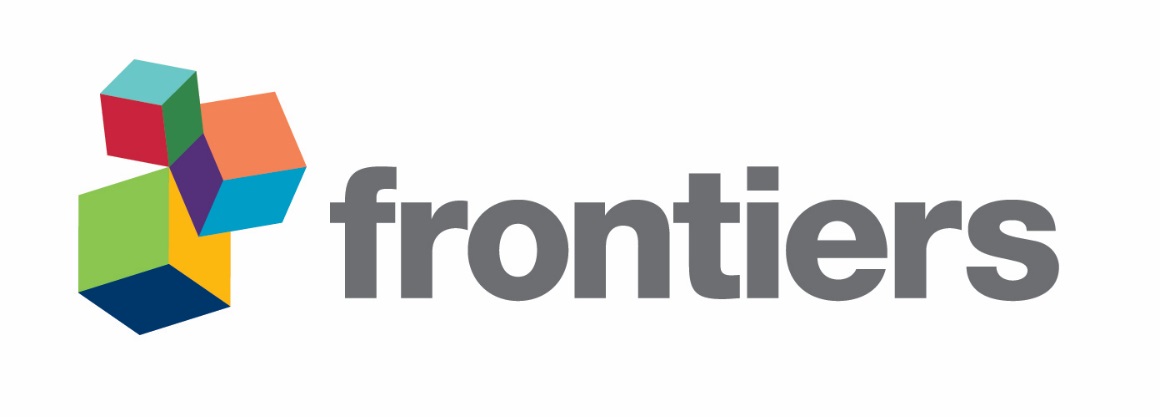
**
